# A problem shared is a problem halved? Comparing burdens arising for family caregivers of patients with disorders of consciousness in institutionalized versus at home care

**DOI:** 10.1186/s40359-018-0272-x

**Published:** 2018-12-14

**Authors:** Inga Steppacher, Johanna Kissler

**Affiliations:** Department of Psychology, University of Bielefeld, Bielefeld, Germany

**Keywords:** Family care givers, Disorders of consciousness, Care burden

## Abstract

**Background:**

Disorders-of-consciousness (DOC) are rare conditions leading to very severe physical and mental disabilities. Providing care for DOC patients has been described as a stressful experience, eroding the physical and psychological health of the caregiver. Different forms of care may have different impacts on the caregivers and institutionalized care has been suggested to have an unburdening effect, but this possibility has never been empirically studied. To address this issue, in this study caregiver-burden between family-caregivers who provide home care themselves and those who have placed their patients in a specialized care unit is compared.

**Method:**

The demographics of the caregivers, life satisfaction, coping strategies, meaning in life, and grief reactions were assessed with questionnaires in 81 long term (m = 7.9 years) caregivers (44 patients in specialized care-units, 37 patients taken care of at home).

**Results:**

Caregiver groups were similar on the vast majority of demographic factors. Remarkably, there were no major differences in self-assessed burden and distress between the two caregiver groups. They both demonstrated generally reduced life satisfaction, were especially dissatisfied with their amount of spare time, and many caregivers in both groups demonstrated long lasting grief reactions, as well as a somewhat enhanced crisis of meaning. However, caregivers with patients in institutionalized care exhibited enhanced self-accusation as well as reduced satisfaction with their own health. Home care caregivers, on the other hand, report below average opportunities to care for themselves.

**Conclusion:**

Surprisingly, placement in institutionalized care in itself does not seem to disburden caregivers as much as expected as the amount of subjective care-giving burden and reported distress is on average similarly high, although profiles differ somewhat according to type of care. Moreover, vast inter-individual variability can be observed. Further research should address the mechanisms that foster positive adjustment and reduce negative impacts for care providers regardless of type of care, enabling the health care system, institutions and self-aid groups alike, to provide more specific support for caregivers by addressing the topics of quality-of-life, own health, self care, and grief reactions.

**Electronic supplementary material:**

The online version of this article (10.1186/s40359-018-0272-x) contains supplementary material, which is available to authorized users.

## Background

To care for an ill or disabled family member is almost always a challenge and has many negative impacts on those providing that care. In fact, in the literature, family caregivers have been called the ‘hidden patients’ [1] or, in cases where the reason for the disability was a traumatic brain injury (TBI), the term ‘head injured family’ has been used [2].

A particular burden arises from the care for patients with disorders of consciousness (DOC). Patients with DOC suffer from one of two syndromes - the unresponsive wakefulness syndrome (UWS; [3]; former vegetative state (VS) [4, 5]) or the minimal consciousness state (MCS; [6]). The first is defined by the total absence of awareness, while cycles of wakefulness (eyes opening) occur. The latter describes a state where patients inconsistently show some marginal signs of awareness. Both syndromes can be stages on the way to recovery, but can also become chronic.

The prevalence of DOC has more often been estimated than measured, and estimates range from 40 and 168 patients per million population for the US [7, 8]. One of the few European studies that actually measured the prevalence of UWS was Stepan et al. [9] who conducted a point prevalence study in Vienna in 2001 which included UWS patients in hospitals and nursing homes. It revealed a prevalence of 19 UWS patients per million. Two more recent nationwide point prevalence studies in Austria also included only institutionalized patients. They found a prevalence of 1 to 2 UWS patients per million population [10] versus 34 UWS patients / 15 MCS patients per million population [11]. Given the wide range of prevalence data, in addition to the fact that neither study included patients being taken care of at home, the actual prevalence of these disorders remains basically unknown. However, it is safe to say that both syndromes, while being relatively rare, have risen in numbers due to advances in intensive care as well as long-term care medicine. This also leaves a rising number of family caregivers of DOC patients whose reality is often described as including two coexisting phenomena: On the one hand, the majority of studies report various negative consequences for the caregivers, such as depression, prolonged grief symptoms [12–14] due to an ambiguous loss experience [15], reduced life satisfaction, health deterioration, anxiety disorders, unsatisfactory family and social relations, as well as financial strains [12–14, 16, 17].

On the other hand, there are also reports of positive experiences, such as feelings of gratification and finding a new purpose in life by adopting the caregiver role [18, 19].

In general, a large body of research has already demonstrated that assuming any caregiving role can result in a state of chronic stress - for DOC caregivers this implies physical and psychological strain over unforeseeable periods of time [12, 13] and evokes the feeling of loss of control since care situations are often highly unpredictable [16]. What is more, the care situation has the potential to create secondary stress in seemingly unrelated life domains, such as work performance and family relations [12, 14]. Lastly, this care situation requires rather constant high levels of vigilance, since patients are unable to communicate specific needs. In fact, caregiving represents chronic stress so well, that caregivers have been used to demonstrate the influence of chronic stress on personal health [20].

In the literature, one solution is primarily suggested to unburden caregivers, namely placing the patient in a nursing home. A considerable amount of research, mostly in association with dementia, has addressed the question of the ‘breaking point’ of family caregivers. This point usually marks the decision of nursing home placement of the patient. Here, high cognitive and functional impairment, as well as declining cognitive functions, high dependency in activities of daily living (ADL), the need to increase time invested in care, as well as urine- and fecal incontinence are strong predictors of nursing home placement [21–24]. In addition, sleep disturbances and communication difficulties also pose major problems for caregivers of elderly patients with multiple diagnoses [25]. In cases of stroke survivors, a further key predictor for nursing home placement is a lack of improvement of the patient [26].

To the best of our knowledge, so far only one study has addressed the question whether caregivers actually feel relieved and less burdened after the nursing home placement of family members. Interestingly, although a significant overall reduction of burden and depressive symptoms was reported, the study showed that wives and daughters continue to feel a clinically relevant burden and husbands still tend to experience clinically relevant depression [27]. This indicates that institutionalized care in itself may not unburden caregivers as much as expected.

In DOC patients, all the factors leading to the overwhelmed breakdown of caregivers in other pathologies, resulting in subsequent nursing home placement of the patient, are present simultaneously and from the beginning. Patients are completely dependent, communication of any kind is impossible, making decisions made for the patient constant guesswork, care is a time-consuming full-time job since the patient is completely dependent in regard to any ADL, urine- and fecal incontinence is given, sleep disturbances are frequent and after the initial months, only very small if any improvement can be expected [4, 6].

It stands to reason that caregivers of these patients experience the significant and often clinically relevant burden and strain that has been reported in previous literature on other disorders. Nevertheless and surprisingly, all extant studies on caregiver burden in DOC actually focus on caregivers that have already made the decision to institutionalize the patient [12–14, 16, 17, 28–31]. Therefore, information about the burden of home care caretakers of DOC patients is largely lacking. This is potentially problematic since, for example in Germany, it is currently politically encouraged to care for DOC patients at home. Additionally, in countries where health insurance / nursing home insurance is not mandatory, it is likely that many families have to care for patients themselves since (specialized) nursing homes are expensive or unavailable. Given that the burden of supposedly already unburdened caregivers of DOC patients still reaches alarmingly high levels [12–14, 16, 17, 28–31], it is all the more important to investigate the burden of home care caretakers of DOC patients. These may be expected to be either generally and significantly more burdened or their burden could present itself in a different areas of focus.

As pointed out before, even caregivers with patients in institutionalized care typically express reduced overall life satisfaction, which is basically a result of reduced physical health, a significant lack of spare time, social isolation and familial [17, 32] and financial strains [13].

We would expect most aspects to be exacerbated by the home care situation, since nursing a bedridden adult patient at home is physically very challenging, adversely affecting physical health. It is also a time-consuming 24/7 job leaving little or no spare time. Concerning social relations, we would assume that caregivers with patients in an institution have the opportunity to engage in social relations with other persons concerned as well as the staff members. This resource is not as readily available for home care caretakers which might enhance the feeling of social isolation. We would further expect that stress, possible overload and the lack of sleep of the primary caregiver further increase family strains in a homecare situation.

Various studies have also indicated that the perceived burden tends to worsen over time. Here, the quality of life usually decreases, whereas emotional burden and family strain increase as the caregiving situation continues [12–14, 17]. However, whether or not the situation continues to deteriorate or improves with time is highly dependent on the adopted coping strategies of the caregivers [12, 16]. Studies have shown that caregivers of DOC patients have trouble adopting adequate and helpful coping strategies, even years after the event [16, 17]. Whether or not both caregiver groups with home care and institutionalized care adopt the same coping strategies or are prone to an overuse of maladaptive strategies is currently unclear.

A rather unique problem reported for DOC caregivers is prolonged grief, due to the ambiguous loss [15] a DOC poses because of its rather unclear ontological state [33]. Here, taking care of the patient at home might lessen the grief reactions by maintaining the housing situation and providing the best possible care. Additionally, homecare might provide caretakers with a more active role and a new purpose in life [19]. This might help home care caretakers to perceive their sacrifices as more meaningful [19, 34] than do caretakers with patients in institutions. Thus, we would expect home care caretakers to draw some psychological benefit from the situation and therefore develop a less pronounced crisis of meaning.

Lastly, for other pathologies as well as DOC, it has been shown that the decision of nursing home placement in itself can be troublesome. Here, caretakers often report very ambivalent emotional responses and feelings of guilt at having abandoned the patient and denied him or her life at home [28, 35].

Despite the severity of the decision on homecare versus institutionalized care placement, there is currently little scientifically informed guidance regarding likely psychological and health consequences of care placement decisions. For the care of DOC patients, to the best of our knowledge, no study exists that compares the psychological and health consequences of each accommodation option. However, such knowledge would increase the chances for appropriate placement of the patient, benefiting both patient and caregiver and helping to avoid unnecessary burden. In our study, we aim to compare two volunteer samples of DOC caregivers, one with their patient in institutionalized care and one caring for the patient at home regarding subjective care burden obtained with several questionnaires, asking for live satisfaction, grief reactions, use of adaptive and maladaptive coping strategies and crisis as well as meaning in life.

## Methods

The study received ethics approval by Bielefeld University’s institutional review board.

### Participants

We included data from 81 long term caregivers of patients with disorders of consciousness. Forty-four patients were taken care of in specialized care units, 37 patients were taken care of at home. All caregivers had been asked to complete a set of questionnaires including socio-demographics of the patient and the caregiver.

Caregivers were recruited between 2015 and 2018 through two specialized care units for patients with DOC (‘Haus Elim’ and ‘Wachkoma Haus Oase’, North-Rhine Westphalia, Germany) and online via several Facebook groups and word-of-mouth recruitment. Furthermore, we advertised the study in the journal ‘NOT’ (Need), a German journal with information for head injured patients and their relatives. NOT offered the link to the online version as well as our email address where interested persons concerned could obtain a paper version of the study. Lastly we spread the online link as well as paper versions via several support groups for caregivers of head injured patients. The return rate for the paper and pencil sets was better than for the online version. We did not keep exact track of the paper and pencil sets but we estimate that about 60% were returned. The online version was called up about 800 times, although the majority only read the welcome site, explaining the purpose of the study. From 144 participants who began with the study, 39 answered the majority of items and could be included into this study. Single missing items were interpolated according to handbook-instructions of the respective questionnaire. If more than one item was missing, the participant was excluded from the analysis of the particular questionnaire. Therefore, participant number varies between questionnaires and is individually specified in the results section.

In both the paper-and-pencil and the online version, the first page explained the purpose of the study, the kind of questions that would follow, the right to abort the study, the anonymity of the data and data storage security. We further assured participants that their data would not be handed over to third parties. Participants agreed either by sending the questionnaires back or by checking the ‘I accept’ box in the online version.

There were no inclusion criteria, other than that participants actually care for a DOC patient since we were interested in general burden of caretaking for all DOC family members.

In our study we referred to DOC patients as patients in a ‘waking coma’ (Wachkoma) which is the colloquial term for UWS in Germany. However, since misdiagnosis between UWS and MCS is very high [36, 37], we expect to have a mixed group of DOC patients.

Of 44 caretakers with patients in institutions, 25 answered the paper and pencil version, 19 used the online version. From 37 home care takers, 18 answered the paper and pencil questionnaire, 19 used the online version of the study (Fisher’s exact test showed no significant association, *p* = .51, indicating that the type of test was randomly distributed between the caregiver groups). Both versions included five different questionnaires in randomized order: demographics, questionnaire about life satisfaction, grief reactions, coping strategies, as well as experienced crisis of meaning. To complete the study, participants needed about an hour. Participants received no payment for the study.

All questionnaires were filled out and returned anonymously.

### Questionnaires

#### The life satisfaction questionnaire

The Fragebogen zur Lebenszufriedenheit (FLZ, [37]) is a widely used standardized questionnaire on life satisfaction in Germany. It provides a measure of the global life satisfaction (main scale) but also allows for differentiation between the following 10 different aspects of life (primary scales): own health, financial situation, spare time, own person, sexuality, social life (friends / acquaintances / relatives), housing conditions, marriage and partnership, relationship with own children, and work. Because not everyone is employed, has children or is in a relationship, the global life satisfaction score is calculated from the first seven scales. Standardized scales are given in Stanines. Internal consistency (Cronbach’s alpha) reaches values between .82 and .95 for the different scales.

#### The questionnaire on coping strategies

The Stressverarbeitungsfragebogen (SVF, [38]) covers a wide range of possible coping strategies used in stressful situations. Two main scales of generally adaptive and maladaptive strategies are formed on the basis of questions about 20 different strategies (primary scales). Sum scores are transformed into T-Values. Internal consistency (Cronbach’s alpha) reaches values between .66 and .96 for the various strategies.

#### The grief questionnaire

The Trauerfragebogen (TF, [39]) is a German adaptation of the Hogan grief reaction checklist [40] which is widely used in research of (prolonged) grief (e.g. [41, 42]). The reference group contains people who suffered the loss of a significant other. They were asked to rate their grief on the various statements of the TF at approximately 3 months after the loss. For the TF, significant differences from the norm group indicate significantly fewer grief symptoms, whereas no difference indicates grief symptoms as severe as in acute grief-stages. For the grief questionnaire, sum scores, means and standard-deviations of the German reference population are reported for the main scale ‘global grief reactions’ as well as for the seven primary scales. Cronbach’s Alpha was calculated and reached .97. For the TF, no norm values are available.

#### The questionnaire on purpose and meaning in life

The Fragebogen zu Lebensbedeutung und Lebenssinn (LEBE, [43]) is an objective method that can be used to achieve a differentiated assessment of meaning in life and crisis of meaning.

It includes the two main scales ‘meaning in life’ (Sinnerfüllung) and ‘crisis of meaning’ (Sinnkrise) which were formed from 26 primary scales regarding goals, plans and intentions. The dimensions are characterized by high stability and generalizability within a person’s life. Sum scores are transformed into T-values, internal consistency (Cronbach’s alpha) reaches values of .65 and .94 for the various primary scales, the main scales reach values of .89 and .93.

### Statistics

To compare the two groups for the results on the various questionnaires, original sum scores were used. Since the literature reports a deterioration of, for example, life satisfaction or a reduced use of adaptive coping strategies for long term caregivers, we included the factor ‘time since event’ as a covariate [12–14, 16, 17]. For every questionnaire main scale, group means were compared with univariate ANCOVAs. Levene’s test was not significant for any of the analyses, indicating similar error variances within the two groups.

In order to explore potential fine-grained group differences in response profiles between these rare and hard to recruit groups without over-inflating the number of t-tests, we further analyse the primary scales of each questionnaire starting with the items with the largest mean differences. Items were sorted according to the size of the group difference and the largest difference was tested for its significance, regardless of the outcome of the main scale ANCOVA.

To compare distributions between groups, Fisher’s exact test was used for 2 × 2 contingency tables, since, for smaller sample sizes, it produces more accurate results than the Chi-Square Test. However, for larger contingency tables, Chi-Square Test was used.

Lastly, the percentage of single participants falling below the normal range of a questionnaire is given to provide information about number of individuals in each group falling into the problematic range.

## Results

Caregiver and patient demographics are summarised in Table 1. Results of the between group comparisons on demographics are also shown in Table 1.

**Table 1:** Caregivers and patient demographics for both accommodation groups

	Home care	Institutionalized care	
N	37	44	
Age of caregiver Mean (SD)	52.35 (14.96)	50.48 (16.64)	t(79) = −0.53 *p* = .60
Gender of caregiver (male / female)	11 / 26	14 / 30	Fisher’s test *p* = 1.00
Taking care of: Parent / Partner / Sibling / Own child / Others/ Missing	3 / 18 / 2 / 12/ 2 / 0	4 / 24 / 3 / 12 / 0 / 1	χ^2^ (4) = 2.77 *p* = .60
Time since event In years, Mean (SD)	9.08 (7.58)	6.70 (4.22)	t(59.6) = 1.71 *p* = .09
Type of event: TBI / hypoxic / others	24 / 11 / 2	21 / 11 / 12	χ^2^(2) = 6.79 *p* = .03
Hours of care per day (SD)	20.12 (7.30)		
Have to get up at night (Yes / No / Missing) How often per Month? (Range)	14 / 20 / 3 30 (2 to 100)		
Patient often otherwise ill / complications? (Yes / No / Missing)	16 / 21 / 0	25 / 18 / 1	Fisher’s test *p* = .26

Sample size (N), mean (M), standard deviation (SD). Significant group differences are highlighted in bold. ‘Patient often otherwise ill / complications’ asked caregivers whether their patient has frequent flues, pneumonia, fevers, decubitus, epileptic seizures, or anything that might intensify care burden. The medical complications were analyzed with Fisher’s test in a 2 × 2 matrix (Yes / No)

Overall, the two groups of caregivers are similar on most parameters. A significant difference in distributions was found only for the type of event initially causing the DOC. This difference appears to result from the fact that rare causes like encephalitis, surgery complication or lightning strike (taken together as ‘others’) are cared for more often in institutions than at home. Additionally, for all care takers together, there are significantly more women than men taking care of family members (χ^2^(1) = 9.99, *p* = .002). However, the sub-groups did not differ in this regard.

**Fig. 1:**
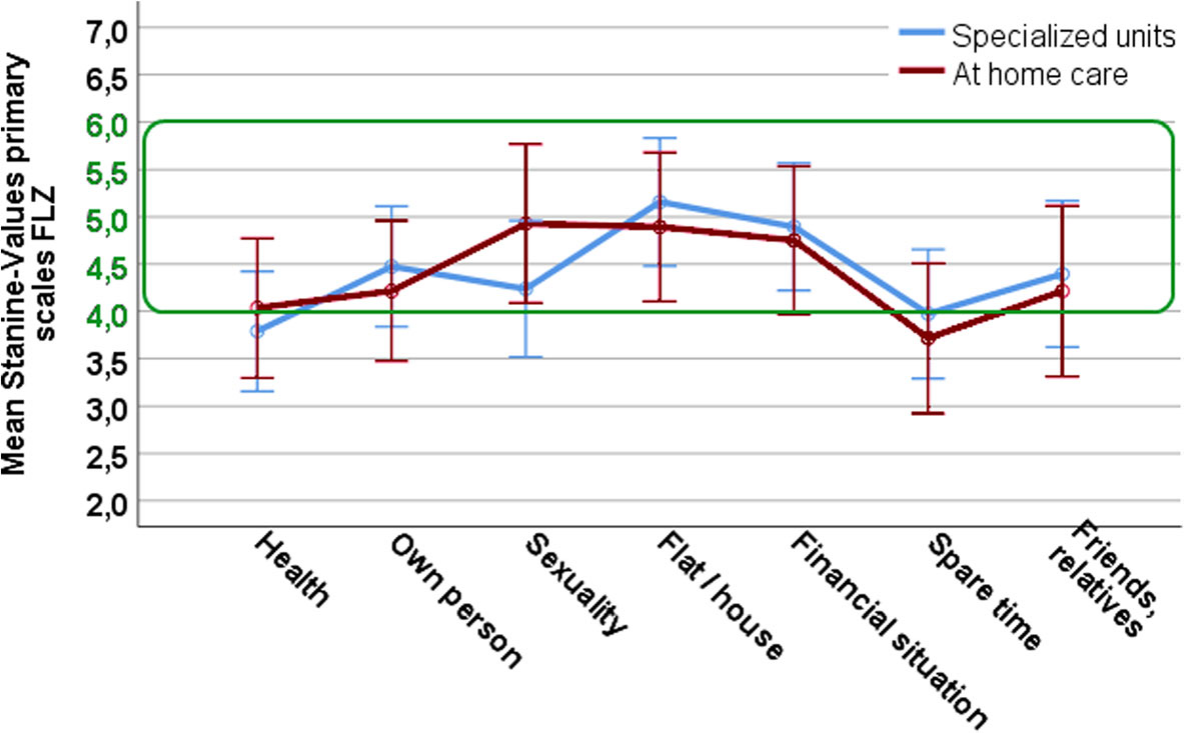
Profile of means on the primary scales for life satisfaction (FLZ) for both caregiver groups. Scores are displayed in Stanine (m = 5, the normal range is marked by the green box. Values under 4 and above 6 are considered as noteworthy). Whiskers display 95% confidence intervals

**Fig. 2:**
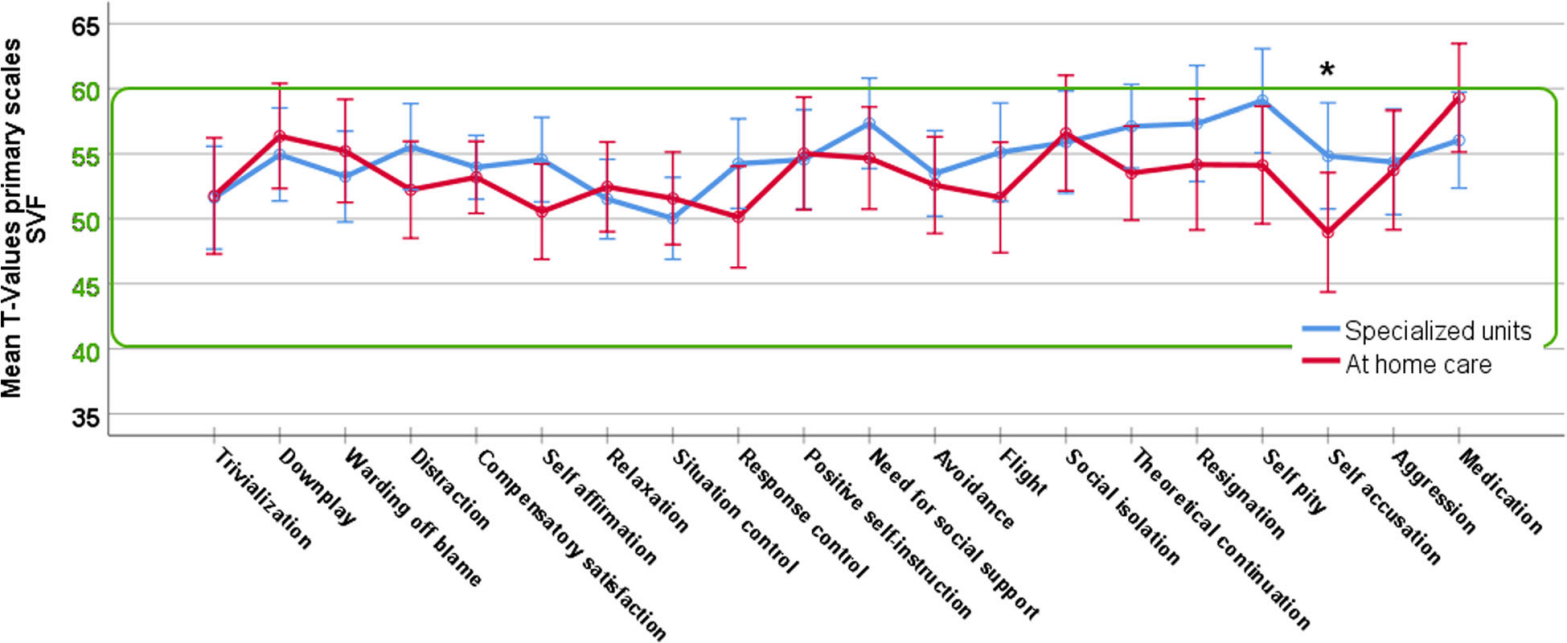
Profile of mean values of the primary scales for coping strategies (SVF) for both caregiver groups. Values are displayed as T-norm-values, normal range is highlighted within the green box. Whiskers display 95% confidence intervals. * indicates significant differences between caregiver groups (* *p* = .05)

**Fig. 3:**
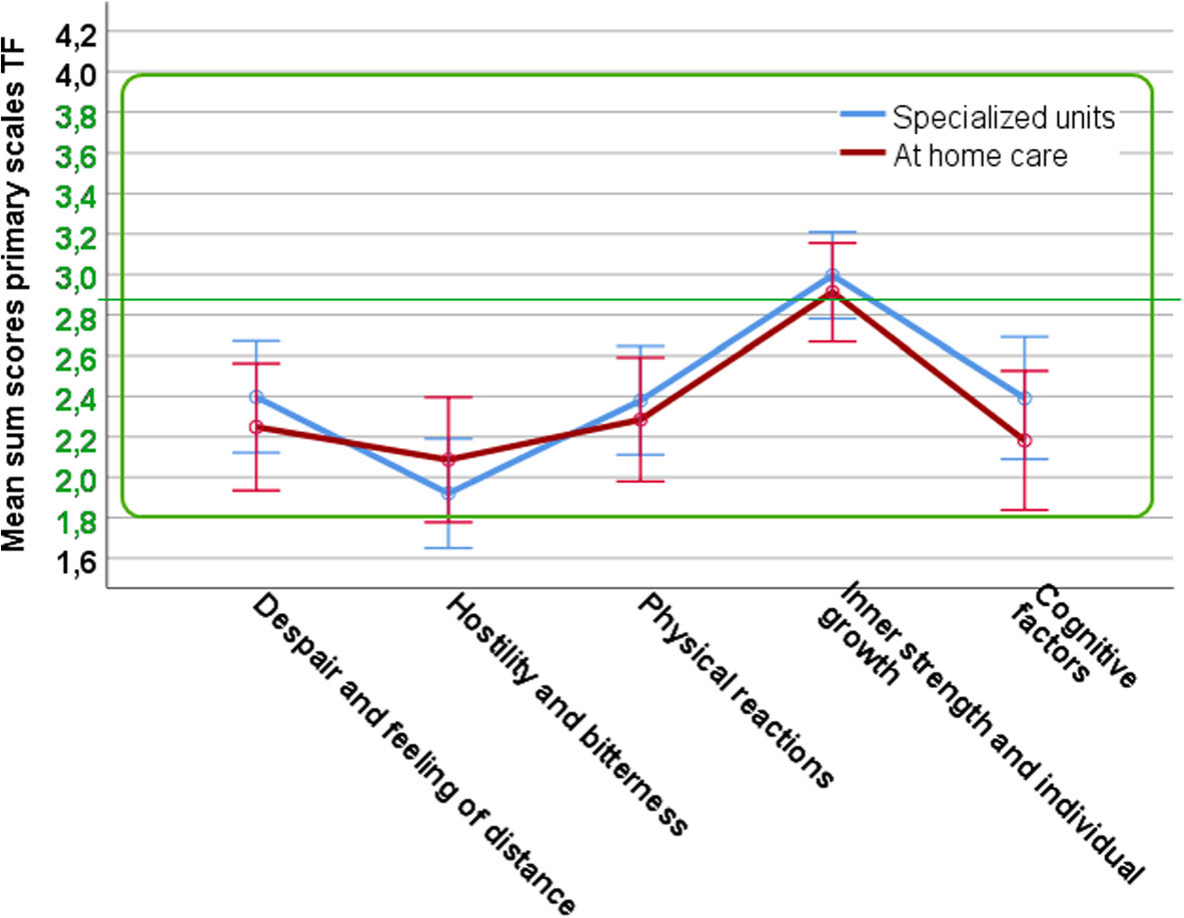
Mean sum score of primary scales of the grief reaction questionnaire (TF). The green midline displays the mean (2.88) of the acutely grieving norm group; the green square highlights plus / minus one SD = 1.08 of the acutely grieving norm group. Whiskers display 95% confidence intervals

**Fig. 4:**
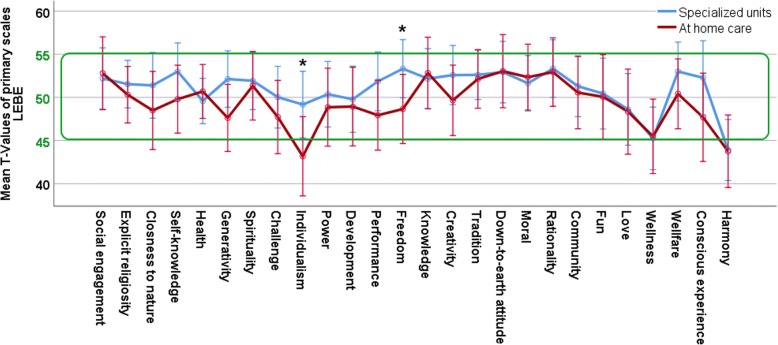
Profile of mean values of the primary scales for meaning in life (LEBE) in both caregiver groups. Values are displayed as T-Norm-Values, normal range is highlighted within the green box (in the LEBE, values of < 45 and > 55 are supposed to be noteworthy [43]). Whiskers display 95% confidence intervals

In general, none of the ANCOVAs performed on the main scales displayed any significant differences between the two groups. Additionally, only live satisfaction was significantly influenced by the time since event (higher live satisfaction after more years as a caretaker). The general results of both groups are given in Table 2. The ANCOVA results are displayed in Table 3. The differences between primary scales are further displayed in Figs 1, 2, 3 and 4.

**Table 2:** Descriptive statistics for the main scales of the questionnaires

Test	Scale	Specialized units			At home care			Norm Values
		*N*	*M*	*SD*	*N*	*M*	*SD*	
FLZ	Global life satisfaction	38	4.01	2.25	28	4.00	2.23	Stanine Norm Range 4–6
SVF	Adaptive strategies	37	54.62	12.28	30	53.46	11.35	T-Values Norm Range 40–60
	Maladaptive strategies	37	58.59	12.63	30	54.11	12.20	T-Values Norm Range 40–60
TF	Global grief score	43	2.42	0.72	33	2.34	0.69	No norm values
LEBE	Meaning in life	42	52.36	9.70	30	51.93	12.35	T-Values Norm Range 40–60
	Crisis of meaning	42	59.95	10.13	30	56.83	8.33	T-Values Norm Range 45–55

Sample size (N) for every questionnaire, mean (M), standard deviation (SD) and norm ranges. Values above or below the norm range would be considered as noticeable findings. Scores deviating from the respective norm population are highlighted in grey. FLZ: Questionnaire on life satisfaction, Scores are reported as Stanine: scores under 4 and over 6 represent deviations from the norm. SVF (questionnaire on coping strategies), LEBE (questionnaire for purpose and meaning in life): Scores are reported as T-norm values. SVF: Scores < 40 and > 60 were considered noteworthy; LEBE: Scores between < 45 and > 55 are considered as noteworthy [43] and highlighted in grey. TF: grief questionnaire, mean value of the norm group: m = 2.88, SD = 1.08

**Table 3:** Results of the ANCOVAs calculated for every questionnaire

	Scale		Df	F	P	η^2^_*p*_
FLZ	Global life satisfaction	Covariate: Time since event	1	4.40	.04	.07
		Group difference	1	0.61	.44	.01
SVF	Adaptive strategies	Covariate: Time since event	1	0.88	.35	.01
		Group difference	1	0.05	.82	.001
	Maladaptive strategies	Covariate: Time since event	1	0.01	.93	.00
		Group difference	1	1.62	.21	.02
TF	Global grief score	Covariate: Time since event	1	1.32	.26	.02
		Group difference	1	0.09	.76	.001
LEBE	Meaning in life	Covariate: Time since event	1	0.08	.77	.001
		Group difference	1	0.10	.76	.001
	Crisis of meaning	Covariate: Time since event	1	0.24	.62	.004
		Group difference	14.4	2.26	.14	.03

FLZ - life satisfaction questionnaire; SVF - questionnaire for coping strategies; TF - grief questionnaire, LEBE - questionnaire for purpose and meaning in life. Levene Test for equality of error variances was never significant. The effect of the covariate ‘time since event’ was only significant for life satisfaction. η^2^_*p*_ = partial eta squared, effect size are mostly small (<.06) and in one case medium (<.14) based on benchmarks suggested by Cohen [50]

For the FLZ, no difference for the global life satisfaction emerges for the two caregiver groups (Table 3). We further compared primary scales to investigate possible specific domains of reduced life satisfaction for any one caregiver group. Results of both groups in the seven primary scales of health, financial situation, spare time, own person, sexuality, social integration, and own housing are displayed in Fig. 1. There were no significant differences between the two groups. Caregivers with patients in institutions display means below the normal range in the sub-categories health (m = 3.71) as well as spare time (m = 3.98). Home care caregivers score borderline on health and below the normal range on spare time (m = 3.78) (see also Additional file 1: Table S1). Lastly, for the life satisfaction scale, in our sample 17 (46%) of home care caregivers and 24 (54.5%) of those with patients in specialized units report low general life satisfaction (scores from 1 to 4 Stanine). Fisher’s exact test indicate that the distribution for both caregiver groups is very similar (*p* = 1.00).

Again, for the scales of adaptive and maladaptive coping strategy use (SVF), there was no difference between the two caregiver groups (Table 3). We further analyse primary scales to inspect specific use of individual coping strategies. In order to minimize t-test, first the scale with the biggest difference between groups was tested. For the SVF, the biggest mean-difference was for self-accusation and it differed significantly between groups t(65) = 2.03, *p* = .047; the second biggest mean difference (self pity) was tested but did not reach significance t(65) = 1.74, *p* = .093. No further t-test was performed. Results and differences between groups are displayed in Fig. 2.

For the SVF, the total numbers of participants scoring below the normal range of adaptive coping strategy use was 3 (6.8%) caregivers for specialized units and 2 (5.4%) caregivers for at home care which did not differ between groups (Fisher’s exact test, *p* = 1.00). The total numbers of caregivers scoring above the normal range of maladaptive coping strategy use were for specialized units 17 (38.6%) and for at home care 11 (29.7%) caregivers which also does not differ between groups (*p* = .47).

There was no difference in the amount of grief symptoms between caregiver groups (Table 3). Also, the primary scales do not differ between groups (Fig. 3). On the single caregiver level, 34 (77.3%) of the caregivers with patients in institutions and 28 (75.7%) home care caregivers reach values indicating severe grief symptoms (above or within one standard deviation of the norm-mean). Fisher’s exact test again shows no differences in the distribution between groups (*p* = .57).

In the LEBE both caregiver groups display enhanced crisis of meaning but did not differ from each other (Table 3). On the primay scale level, a t-tests was performed for the scale with the biggest mean-differnce (individualism) which was significant t(70) = 2.14, *p* = .036. At home caregivers reported significantly reduced individualism. The second biggest mean difference (freedom) was also significant t(70) = 2.04, *p* = .046 and again reduced for at home caregivers. The third biggest difference (generativity) did not reach significance t(70) = 1.77, *p* = .081 (Fig. 4).

On the individual level 32 (76.2%) caregivers of patients in institutions and 19 (63.3%) of home care caregivers reach values of 55 or above on the crisis of meaning scale which is a similar distribution for both groups (Fisher’s exact test, *p* = .30).

Detailed results on each questionnaire (including primary scales) are provided in Additional file 1: Table S1, Additional file 2: Table S2, Additional file 3: Table S3, Additional file 4: Table S4, Additional file 5: Table S5.

No significant differences between the two caregiver groups on main scales were identified. However, both groups displayed reduced life satisfaction and increased crisis of meaning in comparison to the respective norm populations. Both groups also differed significantly from the grieving norm group (caretakers from specialized units t(42) = − 4.24, ***p < .001; home care caretakers t(32) = − 4.46, ***p < .001). This result indicates that caregivers show on average fewer grief symptoms than the acutely grieving norm group. However, as pointed out above, the total number of caregivers still displaying grief reactions like the grieving norm group is still very high.

## Discussion

The present study compared life satisfaction, coping strategies, grief reactions, and purpose and meaning in life of 81 relatives of DOC patients who either cared for their patients at home (*n* = 37) or have them taken care of in a specialized institution (*n* = 44). Despite diligent literature search, we found no other study addressing this question, neither for DOC patients, nor in other pathologies. However, studies on caretaker burden caused by dementia, stroke, and other pathologies assume that nursing home placement is a last resort to unburden overwhelmed caretakers [21–24]. This assumption is already called into question by a study on dementia, which indicated that the unburdening effect of nursing home placement might not be as extensive as suggested [27].

Furthermore, previous research into the burdens of DOC caretakers were performed on caretakers who have already made the decision of nursing home placement. This might reflect the problem of contacting home care caretakers since there are no public registries of any kind for these patients. This makes it extremely difficult to reach a sizeable group of home care caretakers, which might also explain why prevalence studies likewise focus on clinics, rehabilitation centers and nursing homes.

Therefore, our study is, to the best of our knowledge, the first to compare the burdens arising from different types of care for DOC patients by including a home care caregiver group.

In sum, as expected, both caregiver groups differed from the norm values on the following scales: a generally reduced life satisfaction with especially reduced spare time, long lasting grief reactions as well as enhanced crisis of meaning. However, there were very few differences between the two caregiver groups. They scored fairly similarly on all used scales with the three significant exceptions of enhanced self accusation for caretakers with patients in specialized units (Fig. 2) as well as the two findings of reduced individualism and freedom for home caretakers (Fig. 4). There were also slight differences in the satisfaction with one’s own health in the FLZ (with specialized units caretakers being more dissatisfied and the mean of the group being below 4; Fig. 1 and Additional file 1: Table S1) and self accusation from the SVF (again more pronounced for specialized units care takers, Fig. 2 and Additional file 2: Table S2).

In detail, for the life satisfaction scale, in our sample 46% of home care caregivers and 54.5% of those with patients in specialized units reported low general life satisfaction scores. On the different sub-categories of the FLZ, both caregiver groups reported too little leisure time, whereas especially the caregivers with patients in specialized units were more dissatisfied with their own health. The first fits well with the literature where the most common reasons for reduced life satisfaction are the lack of spare-time [17]. However, we initially expected a more pronounced self-reported lack of spare time for home care caregivers since home care-giving is very time consuming. This was not reflected within the FLZ data; however, we found a reduction of the ‘individualism’ and ‘freedom’ scale of the LEBE, basically describing a lack of opportunity to pursue own interests for homecare caretakers. This is in line with the fact that in our sample many home care caretakers described themselves as being around the patient 24 h a day (mean 20 h). On the other hand, caregivers with patients in specialized care institutions were also rather unhappy with their spare time. We expected institutionalized care to unburden family caregivers, providing them with the opportunity to have and enjoy ‘spare-time’. However, it seems that regardless of the accommodation, caregivers spend as much time as possible with the patient. In our data, caregivers, who have the opportunity to do so, even after several years seem to spend the majority of their day with the patients. In our data, two ‘groups’ emerged where unemployed or retired caregivers reported spending the whole day with the patient and those employed reported spending several hours with the patients. This is well in line with results from Leonardi et al. [12] who also reported that about 50% of 487 caregivers with patients in an institution spend three or more hours a day with their patients over a period of many years. So it seems that, as a result, both care-giving groups are more or less equally unhappy with the amount of spare time they have. In our data, as well as in the literature [17], the lack of spare time seems to have a major effect on general life satisfaction. Since it is equally reduced for both caregiver groups, it would be important for family physicians, support groups and institutions to stress the importance of self-care in order to improve life satisfaction and prevent clinically relevant burn-out symptoms.

A second, rather unexpected result, is that especially those caregivers with patients in institutions were unsatisfied with their own health. Actually, we would have expected the home care caregivers to report more physical strains, since the care for an adult bedridden patient is very challenging. However, since we have no means to estimate when the health issues become prominent, this could very well be a pre-existing feature. Maybe caregivers with health concerns were more likely to seek institutionalized care for the patient from the beginning since they might have anticipated to be overstrained with care duty. This notion would be in line with a large study of 580 caregivers of dementia patients, demonstrating that poor health of the caregiver is one of the leading causes of nursing home placement [22]. Here, a longitudinal study, starting even before the accommodation decision for DOC patients, would be very beneficial. It would not only be possible to describe the situations which actually lead to the choice of care in more detail, it would also be informative to see how satisfied caregivers are with that decision in the long run. Additionally, it would be possible to analyze the situational and personal factors predicting this satisfaction, enabling clinicians to improve their advisory service.

On the other hand, neither group reported to be unsatisfied with their social integration, which was suggested to be one of the major problems in previous literature [17, 32]. Here, we expected caregivers to have a better social integration if their patient is taken care of in an institution, since institutions provide the opportunity for social contacts with others in the same situation. Of course, a support group would have the same effect for home care caregivers. However, as a limitation of this study, we did not ask home care caregivers if they attend a support group but since we spread the online-version of this study, besides other ways, also via support group websites, it is reasonable to assume that at least some of the home care caregivers are members of such groups. So, the absence of any differences regarding social integration may be an effect of a self-selection bias where the really isolated home care providers would not know about the study and/ or did not find the time to answer our questions.

The questionnaire on coping strategies (SVF) revealed no difference between the two caregiver groups in the use of general adaptive and maladaptive strategies. Furthermore, on average, no category of strategies was used more or less frequently than by the norm group. From the primary scales, the most frequently used maladaptive strategy was self-accusation, which actually differs significantly in use between the two caregiver groups. In tendency, there was also a difference in the occurrence of self-pity which might be related to self-accusation. It was again more common for caregivers with patients in specialized institutions. Stern et al., [31], as well as Cipolletta and colleagues [28] also reported, that families tend to react with serious feelings of guilt, if the patient is seen as ‘abandoned’ (taken care of in an institution). This guilt could be the reason for enhanced self-accusation in caregivers whose patients are taken care of in a specialized unit. Additionally, the same mechanisms could be the reason for the enhanced self pity: Although research on self-pity is scarce, Grunert for instance [44] pointed out that self-pity could be interpreted as a self-defense mechanism. It occurs whenever a person feels shame and aggression but cannot act on it. It is argued that guilt can lead to (self)-aggression (which has been reported to occur in DOC caregivers with patients in institutions [31]) as well as self-accusation [45], which in turn could transform into self-pity - a feeling easier to cope with and, given the caregivers’ situation, maybe more socially accepted. Initially, self-pity might very well evoke empathy from others [46]; however, a pervasive display of self-pity will not [47]. So, self-pity could, in the long run, contribute to social isolation and family strains.

Here, the consultation and approval of an accepted authority seems to be one way to counteract the guilt related with nursing home placement. It seems that the consultation with an authority, usually a medical doctor, confers some kind of legitimization to the decision. For instance, caregivers of elderly persons who made the decision of nursing home placement and did not discuss it with a professional viewed their decisions most negatively [35]. To have the approval of a professional medical practitioner seems to somewhat prevent the ambivalent emotional feelings toward this decision and can minimize the feelings of guilt [35], which otherwise can lead to long lasting maladaptive behavior of the caretaker, such as making up to the patient by spending every available time at the institution at the expense of own health, potentially even leading to self-abandonment [28].

On the positive side, very few caregivers scored below the norm in the use of adaptive strategies. So, a general lack of adaptive strategies in long term caregivers of UWS patients in institutionalized care, as reported by Cipolletta et al. [16] cannot be confirmed in our sample.

For the grief questionnaire (TF), the sum scores of the caregiver groups indicated that both groups grieve less than the acutely grieving norm group. Yet, on the single caregiver level, it becomes obvious that most of the caregivers (34 (77.3%) with patients in institutions and 28 (75.7%) home-care caregivers) still suffer from severe grief symptoms (above or within one standard deviation of the norm-mean). This result is in line with Boss [15], describing the ambiguous loss as a long lasting state, which is hard to bear. Arising from this situation are mixed feelings of mourning and hope, with the latter preventing the former from fully being processed and adapted to. However, on the positive side, both caregiver groups reported high values on the scale ‘inner strength and personal growth’. This scale contains 10 items like ‘I can show more compassion to others now’, ‘I am more tolerant to others now’, or ‘I reached a point where I can begin to let go of some of the grief’. As Christopher put it, a significant experience has the potential to change a person [48] and although most of these changes might be for the worse, there could also be changes in the world view of the person, that might foster individual growth. In fact, the positive potential that could arise from a situation like care-giving [18], receives far too little attention within the research of consequences of care-giving so far.

In the LEBE, we found high values in crisis of meaning for caretakers, as expected slightly more pronounced for those with patients in specialized institutions. Here, we assume that taking care of the patient at home can actually somewhat protect from the crisis by offering some new meaning in life. For example, caretakers of Alzheimer’s patients sometimes report finding a new meaning in life through the feeling of being needed by the patient [19, 34]. Therefore, the process of redefining the meaning in life seems to be somewhat more challenging for caretakers with patients in specialized institutions. This demonstrates that even years after the event caretakers struggle to ascribe meaning to the situation. This struggle seems more pronounced for caretakers who do not experience themselves as crucial for the wellbeing of the patient. Additionally, since the patients are incapable of demonstrating whether or not they are aware of, or grateful for, their caretakers’ sacrifices, family members are more prone to doubt the usefulness of all their efforts. That home care caretakers struggle a bit less with a crisis of meaning might also reflect itself in the result that in our sample, with time, life satisfaction actually increases significantly for this group but not for caregivers with patients in institutions. This indicates that institutions and support groups alike should address these questions in order to give caregivers a space to discuss their doubts, which might help them to find a new and maintainable balance between their own life and patient care.

In general, a positive result of this study is, that, for the whole group of caregivers, the situation seems to become more bearable over time. Life satisfaction actually increases as the caregiving continues and does not deteriorate, as reported in other studies. Whether this is because our sample manages to juggle a satisfying social live with care duty or because family strains and financial problems are not as pronounced in our sample as in other samples [12, 14, 17] presently cannot be answered .

Lastly, it is important to note that there are some limitations to this study. For one, it is very difficult to recruit homecare caretakers, which might explain why no previous research has studied them so far. As described earlier, we spread the link to the online version of the study via Facebook-Groups, support groups, the journal ‘Not’ and clinical contacts. However, this implies that we might have only reached those that are actively searching information in the internet or are organized in some kind of DOC (or similar) support group which might have provided them with the online link or the paper and pencil version of our study. This may have led to a selective group of DOC caretakers, where we did not reach those that are truly isolated. Additionally, the length of the questionnaires might have further fostered this effect. It might have added to a selection bias by only including those who were willing to spend about an hour of their time on our research. This might have excluded the most heavily burdened caretakers, regardless of the type of care and might help explain the generally moderate findings of our study. Therefore, further research should aim to find a different method to reach especially those caregivers ‘buried’ in their daily duties in order to include them into the study to specify caregiver burden. Furthermore, group sizes might have limited the statistical power to detect differences between groups, although effect sizes for the between-group comparisons were rather low, suggesting that in the absence of systematic distortions in the two samples, larger sample sizes alone may not change the pattern of results. Based on the present results, future studies could focus on fewer problem areas of interest and implement a shorter survey in order to prevent dropouts and therefore reach higher sample sizes. In fact, several participants send us feedback via Facebook, complaining that standardized questionnaires did not adequately capture their situation, stating that they discontinued the survey for this reason. Here, structured or semi-structured telephone interviews have proven to be effective tools to reach large cohorts of participants [49]. In larger cohorts it would also be very interesting to further distinguish between the reported burdens for different family members. It is intuitive to assume that the felt burden would differ for a spouse caring for his/her wife/husband, compared to parents caring for a child. However, due to current sample sizes, such a distinction could not have been made in this study.

Given the possible self-selection bias of our study, our results could actually underestimate the burden of both caregiver groups. Such an effect could be more pronounced for home care caretakers. On the positive side, results demonstrate that, in relation to overall prevalence estimates, a considerable number of both homecare and institutional caretakers of DOC patients could be reached, and many of those manage to adapt to the situation and in many respects maintain or regain psychological and physical health. In particular, the study suggests that home care does not need to be a dead-end leading to physical and mental breakdown. Future studies should further specify the mechanisms that promote individual well-being in caretakers and help prevent dysfunctions that can quickly become costly on an individual and societal level.

## Conclusion

To sum up, in our sample both caregiver groups demonstrated low average life satisfaction, experience some crisis of meaning and show some persistent grief reactions. Both groups make use of a wide range of coping strategies.

On the sub-scale level, it becomes obvious that whether a patient is cared for at home or in a professional institution produced slightly different burden profiles, but remarkably, the relatives of patients in specialized institutions seem, overall, somewhat worse off. In particular, the crisis of meaning is somewhat higher and the guilt of having ‘abandoned’ the patient, showing itself in more frequent self accusation, as well as self pity, seems to be an obstacle.

These results demonstrate that, no matter the decision of home-care or institutionalized care, support groups and / or institutions should be encouraged to address the topics of life quality, self care of caregivers, and prolonged grief symptoms. Furthermore, in the case of institutionalized care, it might also be advisable for medical professionals to discuss the decision and the reasons to institutionalize patients with caretakers before the nursing home placement. This discussion should be timed well before clinical discharge or the end of the rehabilitation period. Additionally, it might commend itself to offer the opportunity for caregivers to speak about the taboo feelings of shame and guilt as well as doubts about their new role in the patient’s life, and in their own for that matter, in order to confront the problems arising from them before they can lead to clinically relevant consequences.

## Additional files

Additional file 1: **Table S1.** Results of the life satisfaction scale (FLZ) for family caregivers with patients in specialized units and taken care of at home**.** Note: Sample size (N), mean (M) and standard deviation (SD). Scores are reported as Stanine. Scores under 4 and over 6, as deviations from the norm, are highlighted in grey. (DOCX 12 kb)

Additional file 2: **Table S2.** Results of the coping strategies questionnaire (SVF) for family caregivers with patients in specialized units and taken care of at home. Note: Mean (M), standard deviation (SD) und t-statistics (T, df, p; * *p* < .05). Scores are reported as T-norm-scores. Significant differences between groups are highlighted in bold. (DOCX 14 kb)

Additional file 3: **Table S3.** Results of the grief questionnaire (TF): Comparison of caregivers with patients in specialized units and the grieving norm group. Note: Displayed are sample size (N), mean (M), standard deviation (SD) und t-statistics (T, df, p; * *p* < .05, ** *p* < .01, ***p < .001). Significant differences between this caregiver group and the norm are highlighted in bold. Note that in this case the non-significant differences are of more interest, since they represent grief-scores in caregivers that are comparable with acute severe mourning. (DOCX 13 kb)

Additional file 4: **Table S4.** Results of the grief questionnaire (TF): Comparison of caregivers taking care of patients at home and the grieving norm group. Note: Displayed are sample size (N), mean (M), standard deviation (SD) und t-statistics (T, df, p; * *p* < .05, ** *p* < .01, ***p < .001). Significant differences between this caregiver group and the norm are highlighted in bold. Note that in this case the non-significant differences are of more interest, since they represent grief-scores in caregivers years after the event comparable with acute severe mourning. (DOCX 13 kb)

Additional file 5: **Table S5.** Results for caregiver groups for the questionnaire for purpose and meaning in life (LEBE). Note: Scores between < 45 and > 55 are considered noteworthy. Displayed are mean (M) and standard deviation (SD). Deviations from the norm are highlighted in gray. (DOCX 15 kb)

## Abbreviations

DOC: disorders of consciousness
UWS: unresponsive wakefulness syndrome
VS: vegetative state
MCS: minimally consciousness state
ANOVA: analysis of variance
TBI: traumatic brain injury
ADL: activities of daily living

## References

[1] Chwalisz K (1992). Perceived stress and caregiver burden after brain injury: a theoretical integration. Rehabilitation Psychology.

[2] Brooks DN (1991). The head-injured family. J Clin Exp Neuropsychol.

[3] Laureys S, Celesia GG, Cohadon F, Lavrijsen J, León-Carrión J, Sannita WG (2010). Unresponsive wakefulness syndrome: a new name for the vegetative state or apallic syndrome. BMC Med.

[4] The Multi-Society Task Force on PVS (1994). Medical aspects of the persistent vegetative state (1). N Engl J Med.

[5] The Multi-Society Task Force on PVS (1994). Medical aspects of the persistent vegetative state (2). The Multi-Society Task Force on PVS. N Engl J Med.

[6] Giacino JT, Ashwal S, Childs N, Cranford R, Jennett B, Katz DI (2002). The minimally conscious state: definition and diagnostic criteria. Neurology.

[7] Beaumont JG, Kenealy PM (2005). Incidence and prevalence of the vegetative and minimally conscious states. Neuropsychological rehabilitation.

[8] Jennett B. The vegetative state: J. Neurol. Neurosurg. Psychiatry. 2002;355–357 10.1136/jnnp.73.4.355 PMC1738081 12235296

[9] Stepan C, Haidinger G, Binder H (2004). Prevalence of persistent vegetative state/apallic syndrome in Vienna. Eur J Neurol.

[10] van Erp WS, Lavrijsen JCM, Vos PE, Bor H, Laureys S, Koopmans RTCM (2015). The vegetative state: prevalence, misdiagnosis, and treatment limitations. J Am Med Dir Assoc.

[11] Donis J, Kräftner B (2011). The prevalence of patients in a vegetative state and minimally conscious state in nursing homes in Austria. Brain Inj.

[12] Leonardi M, Giovannetti AM, Pagani M, Raggi A, Sattin D, Patients (2012). Burden and needs of 487 caregivers of patients in vegetative state and in minimally conscious state: results from a national study. Brain Inj.

[13] Moretta P, Estraneo A, de Lucia L, Cardinale V, Loreto V, Trojano L (2014). A study of the psychological distress in family caregivers of patients with prolonged disorders of consciousness during in-hospital rehabilitation. Clin Rehabil.

[14] Bastianelli A, Gius E, Cipolletta S (2016). Changes over time in the quality of life, prolonged grief and family strain of family caregivers of patients in vegetative state: a pilot study. J Health Psychol.

[15] Boss P. Ambiguous loss: Learning to live with unresolved grief: New York. Harvard University Press; 2009.

[16] Cipolletta S, Gius E, Bastianelli A (2014). How the burden of caring for a patient in a vegetative state changes in relation to different coping strategies. Brain Inj.

[17] Chiambretto P, Ferrario SR, Zotti AM (2001). Patients in a persistent vegetative state: caregiver attitudes and reactions. Acta Neurol Scand.

[18] Schulz R, Sherwood PR (2008). Physical and mental health effects of family caregiving. J Soc Work Educ.

[19] Zarit SH (2012). Positive aspects of caregiving: more than looking on the bright side. Aging Ment Health.

[20] Vitaliano PP, Zhang J, Scanlan JM (2003). Is caregiving hazardous to one's physical health? A meta-analysis. Psychol Bull.

[21] Annerstedt L, ElmstÅhl S, Ingvad B, Samuelsson S-M (2000). An analysis of the caregiver's burden and the" breaking-point" when home care becomes inadequate. Scand J Public Health.

[22] Buhr GT, Kuchibhatla M, Clipp EC (2006). Caregivers' reasons for nursing home placement: clues for improving discussions with families prior to the transition. The Gerontologist.

[23] Cameron JI, Stewart DE, Streiner DL, Coyte PC, Cheung AM (2014). What makes family caregivers happy during the first 2 years post stroke?. Stroke.

[24] Cohen CA, Gold DP, Shulman KI, Wortley JT, McDonald G, Wargon M (1993). Factors determining the decision to institutionalize dementing individuals:a prospective study. The Gerontologist.

[25] Kesselring A (2001). Emotional and physical demands on caregivers in home care to the elderly in Switzerland and their relationship to nursing home admission. Eur J Public Health.

[26] Mackenzie A, Greenwood N (2012). Positive experiences of caregiving in stroke: a systematic review. Disabil Rehabil.

[27] Gaugler JE, Mittelman MS, Hepburn K, Newcomer R (2010). Clinically significant changes in burden and depression among dementia caregivers following nursing home admission. BMC Med.

[28] Cipolletta S, Pasi M, Avesani R (2016). Vita tua, mors mea: the experience of family caregivers of patients in a vegetative state. J Health Psychol.

[29] Chiambretto P, Moroni L, Guarnerio C, Bertolotti G, Prigerson HG (2010). Prolonged grief and depression in caregivers of patients in vegetative state. Brain Inj.

[30] Sagmüller P (2011). Die subjektiven Belastungen von Pflegepersonen bei der Betreuung von Menschen im Wachkoma in der stationären Langzeitbetreuung: uniwien.

[31] Stern JM, Sazbon L, Becker E, Costeff H (1988). Severe behavioural disturbance in families of patients with prolonged coma. Brain Inj.

[32] Vogler J, Klein AM, Bender A. Long-term health-related quality-of-life in patients with acquired brain injury and their caregivers. Brain Inj. 2014;28(11):1381–8. 10.3109/02699052.2014.919536 24945467

[33] Gray K, Knickman TA, Wegner DM (2011). More dead than dead: perceptions of persons in the persistent vegetative state. Cognition.

[34] Semiatin AM, O'Connor MK (2012). The relationship between self-efficacy and positive aspects of caregiving in Alzheimer's disease caregivers. Aging Ment Health.

[35] Lundh U, Sandberg J, Nolan M (2000). ‘I don’t have any other choice’: spouses’ experiences of placing a partner in a care home for older people in Sweden. J Adv Nurs.

[36] Andrews K, Murphy L, Munday R, Littlewood C (1996). Misdiagnosis of the vegetative state: retrospective study in a rehabilitation unit. BMJ.

[37] Childs NL, Mercer WN (1996). Misdiagnosing the persistent vegetative state. Misdiagnosis certainly occurs. BMJ.

[38] Wilhelm J, Erdmann G, Boucsein W. Stressverarbeitungsfragebogen:(SVF). Verlag für Psychologie Hogrefe, 1985.

[39] Weiser P. Trauerreaktionen: eine empirische Studie zur Untersuchung von Trauerreaktionen in der Bundesrepublik Deutschland unter Verwendung einer deutschen Version der Hogan Grief Reaction Checklist: Johannes-Gutenberg-Universität, Interdisziplinärer Arbeitskreis Thanatologie; 2003.

[40] Hogan NS, Greenfield DB, Schmidt LA (2001). Development and validation of the Hogan grief reaction checklist. Death Stud.

[41] Mularski RA, Dy SM, Shugarman LR, Wilkinson AM, Lynn J, Shekelle PG (2007). A systematic review of measures of end-of-life care and its outcomes. Health Serv Res.

[42] Sanders S, Ott CH, Kelber ST, Noonan P (2008). The experience of high levels of grief in caregivers of persons with Alzheimer's disease and related dementia. Death Stud.

[43] Schnell T, Becker P (2007). LeBe. Fragebogen Zu Lebensbedeutungen Und Lebenssinn.

[44] Grunert U (1988). Self dialog in self pity. Psyche (Stuttg).

[45] Wilson SL (1985). The self-pity response: a reconsideration. Progress in self-psychology.

[46] Milrod D (1972). Self-pity, self-comforting, and the superego. Psychoanal Study Child.

[47] Charmaz KC. The social construction of self-pity in the chronically ill. Studies in symbolic interaction; 1980.

[48] Christopher M (2004). A broader view of trauma: a biopsychosocial-evolutionary view of the role of the traumatic stress response in the emergence of pathology and/or growth. Clin Psychol Rev.

[49] Lai DWL. Effect of financial costs on caregiving burden of family caregivers of older adults. SAGE Open. 2012;2(4):2158244012470467.

[50] Cohen J (1988). Statistical power analysis for the behavioral sciences.

