# Supplementary material for: A problem shared is a problem halved? Comparing burdens arising for family caregivers of patients with disorders of consciousness in institutionalized versus at home care
Source: BMC Psychol. 2018 Dec 14;6:58. doi: 10.1186/s40359-018-0272-x (PMC6295043; doi:10.1186/s40359-018-0272-x)
Supplement: Supplementary file 1 — Table S1. Results of the life satisfaction scale (FLZ) for family caregivers with patients in specialized units and taken care of at home. Note: Sample size (N), mean (M) and standard deviation (SD). Scores are reported as Stanine. Scores under 4 and over 6, as deviations from the norm, are highlighted in grey. (DOCX 12 kb) [file 40359_2018_272_MOESM1_ESM.docx]

**S1 Table. Results of the life satisfaction scale (FLZ) for family caregivers with patients in specialized units vs. taken care of at home**

|  | Specialized units | | | At home care | | |
| --- | --- | --- | --- | --- | --- | --- |
|  | *N* | *M* | *SD* | *N* | *M* | *SD* |
| Main scale |  |  |  |  |  |  |
| Global satisfaction | 38 | 4.10 | 2.22 | 28 | 4.00 | 2.28 |
| Primary scales |  |  |  |  |  |  |
| Health | 41 | 3.71 | 1.87 | 33 | 4.33 | 2.10 |
| Financial situation | 41 | 4.80 | 2.00 | 33 | 4.81 | 2.05 |
| Spare time | 40 | 3.98 | 2.02 | 32 | 3.78 | 2.03 |
| Own person | 41 | 4.44 | 2.15 | 32 | 4.28 | 1.73 |
| Sexuality | 39 | 4.15 | 2.17 | 28 | 4.92 | 2.34 |
| Friends, acquaintances, relatives | 41 | 4.37 | 2.20 | 30 | 4.30 | 2.57 |
| Flat / house | 41 | 5.05 | 2.20 | 30 | 4.97 | 1.96 |

Note: Sample size (N), mean (M) and standard deviation (SD). Scores are reported as Stanine. Scores under 4 and over 6, as deviations from the norm, are highlighted in grey.
