# Supplementary material for: A problem shared is a problem halved? Comparing burdens arising for family caregivers of patients with disorders of consciousness in institutionalized versus at home care
Source: BMC Psychol. 2018 Dec 14;6:58. doi: 10.1186/s40359-018-0272-x (PMC6295043; doi:10.1186/s40359-018-0272-x)
Supplement: Supplementary file 2 — Table S2. Results of the coping strategies questionnaire (SVF) for family caregivers with patients in specialized units and taken care of at home. Note: Mean (M), standard deviation (SD) und t-statistics (T, df, p; * p < .05). Scores are reported as T-norm-scores. Significant differences between groups are highlighted in bold. (DOCX 14 kb) [file 40359_2018_272_MOESM2_ESM.docx]

**S2 Table. Results of the coping strategies questionnaire (SVF 120) for family caregivers with patients in specialized units and taken care of at home.**

|  | Specialized units (n= 37) | | At home care (n= 30) | | |
| --- | --- | --- | --- | --- | --- |
|  | *M* | *SD* | | *M* | *SD* |
| Main scales |  |  | |  |  |
| Adaptive strategies | 54.62 | 12.28 | | 53.46 | 11.35 |
| Maladaptive strategies | 58.59 | 12.63 | | 54.11 | 12.20 |
| Primary scales |  |  | |  |  |
| Trivialization | 51.62 | 12.29 | | 51.73 | 11.55 |
| Downplay | 54.94 | 12.58 | | 56.37 | 8.15 |
| Warding off blame | 53.25 | 10.97 | | 55.21 | 10.08 |
| Distraction | 55.32 | 10.05 | | 52.16 | 9.82 |
| Compensatory satisfaction | 53.97 | 7.75 | | 53.22 | 6.99 |
| Self affirmation | 54.56 | 9.58 | | 50.73 | 10.18 |
| Relaxation | 51.51 | 9.08 | | 52.51 | 9.47 |
| Situation control | 50.03 | 10.19 | | 51.19 | 8.91 |
| Response control | 54.24 | 10.54 | | 49.83 | 10.46 |
| Positive self-instruction | 54.55 | 12.07 | | 54.42 | 11.37 |
| Need for social support | 57.34 | 9.79 | | 54.84 | 11.32 |
| Avoidance | 53.49 | 11.13 | | 52.60 | 8.25 |
| Flight | 55.12 | 12.21 | | 51.87 | 10.32 |
| Social encapsulation | 55.88 | 13.05 | | 56.73 | 10.21 |
| Theoretical continuation | 57.11 | 9.56 | | 53.23 | 9.99 |
| Resignation | 57.32 | 14.61 | | 54.03 | 11.90 |
| Self-pity | 59.08 | 12.37 | | 54.02 | 11.71 |
| Self-accusation | **54.83** | **12.20** | | **48.69** | **12.51** |
| Aggression | 54.37 | 12.53 | | 53.75 | 11.91 |
| Medication | 55.86 | 11.58 | | 59.23 | 10.41 |

Note: Mean (M), standard deviation (SD). Scores are reported as T-norm-scores. Significant differences between groups are highlighted in bold.
