# Supplementary material for: A problem shared is a problem halved? Comparing burdens arising for family caregivers of patients with disorders of consciousness in institutionalized versus at home care
Source: BMC Psychol. 2018 Dec 14;6:58. doi: 10.1186/s40359-018-0272-x (PMC6295043; doi:10.1186/s40359-018-0272-x)
Supplement: Supplementary file 3 — Table S3. Results of the grief questionnaire (TF): Comparison of caregivers with patients in specialized units and the grieving norm group. Note: Displayed are sample size (N), mean (M), standard deviation (SD) und t-statistics (T, df, p; * p < .05, ** p < .01, ***p < .001). Significant differences between this caregiver group and the norm are highlighted in bold. Note that in this case the non-significant differences are of more interest, since they represent grief-scores in caregivers that are comparable with acute severe mourning. (DOCX 13 kb) [file 40359_2018_272_MOESM3_ESM.docx]

**S3 Table. Results of the grief questionnaire (TF): Comparison of caregivers with patients in specialized units and the grieving norm group.**

|  | Specialized units  (n=43) | | | | Norm | | t-Test | | |
| --- | --- | --- | --- | --- | --- | --- | --- | --- | --- |
|  | | *M* | *SD* | *M* | | *SD* | T | Df | *p* |
| Global grief score | | 2.42 | 0.72 | 2.88 | | 1.08 | -4.24 | 42 | **.001**** |
| Primary scales | |  |  |  | |  |  |  |  |
| Despair and a feeling of distance | | 2.39 | 0.96 | 3.36 | | 1.08 | -6.57 | 42 | **.000**** |
| Hostility and bitterness | | 1.92 | 0.83 | 2.12 | | 1.29 | -1.58 | 42 | .121 |
| Physical reactions | | 2.40 | 0.93 | 2.04 | | 1.05 | 2.39 | 42 | .021 |
| Inner strength and individual growth | | 3.00 | 0.68 | 3.15 | | 1.17 | -1.50 | 42 | .141 |
| Cognitive factors | | 2.40 | 1.04 | 2.62 | | 0.79 | -1.44 | 42 | .156 |

Note: Displayed are sample size (N), mean (M), standard deviation (SD) und t-statistics (T, df, Bonferroni corrected p-value = .01; *p< .01, **p< .001). Significant differences between this caregiver group and the norm are highlighted in bold. Note that in this case the non-significant differences are of more interest, since they represent grief-scores in caregivers that are comparable with acute severe mourning.
