# Supplementary material for: A problem shared is a problem halved? Comparing burdens arising for family caregivers of patients with disorders of consciousness in institutionalized versus at home care
Source: BMC Psychol. 2018 Dec 14;6:58. doi: 10.1186/s40359-018-0272-x (PMC6295043; doi:10.1186/s40359-018-0272-x)
Supplement: Supplementary file 4 — Table S4. Results of the grief questionnaire (TF): Comparison of caregivers taking care of patients at home and the grieving norm group. Note: Displayed are sample size (N), mean (M), standard deviation (SD) und t-statistics (T, df, p; * p < .05, ** p < .01, ***p < .001). Significant differences between this caregiver group and the norm are highlighted in bold. Note that in this case the non-significant differences are of more interest, since they represent grief-scores in caregivers years after the event comparable with acute severe mourning. (DOCX 13 kb) [file 40359_2018_272_MOESM4_ESM.docx]

**S4 Table. Results of the grief questionnaire (TF): Comparison of caregivers taking care of patients at home and the grieving norm group.**

|  | At home care  (n=33) | | | Norm | t-Test | | |
| --- | --- | --- | --- | --- | --- | --- | --- |
|  | *M* | *SD* | *M* | *SD* | T | Df | *p* |
| Global grief score | 2.34 | 0.69 | 2.88 | 1.08 | -4.46 | 32 | **.000**** |
| Primary scales |  |  |  |  |  |  |  |
| Despair and a feeling of distance | 2.24 | 0.83 | 3.36 | 1.08 | -7.74 | 32 | **.000**** |
| Hostility and bitterness | 2.09 | 0.97 | 2.12 | 1.29 | -0.20 | 32 | .844 |
| Physical reactions | 2.28 | 0.81 | 2.04 | 1.05 | 1.73 | 32 | .093 |
| Inner strength and individual growth | 2.91 | 0.74 | 3.15 | 1.17 | -1.90 | 32 | .072 |
| Cognitive factors | 2.18 | 0.92 | 2.62 | 0.79 | -2.74 | 32 | **.010*** |

Note: Displayed are sample size (N), mean (M), standard deviation (SD) und t-statistics (T, df, Bonferroni corrected p-value = .01; * p< .01, **p< .001). Significant differences between this caregiver group and the norm are highlighted in bold. Note that in this case the non-significant differences are of more interest, since they represent grief-scores in caregivers years after the event comparable with acute severe mourning.
