# Supplementary material for: A problem shared is a problem halved? Comparing burdens arising for family caregivers of patients with disorders of consciousness in institutionalized versus at home care
Source: BMC Psychol. 2018 Dec 14;6:58. doi: 10.1186/s40359-018-0272-x (PMC6295043; doi:10.1186/s40359-018-0272-x)
Supplement: Supplementary file 5 — Table S5. Results for caregiver groups for the questionnaire for purpose and meaning in life (LEBE). Note: Scores between < 45 and > 55 are considered noteworthy. Displayed are mean (M) and standard deviation (SD). Deviations from the norm are highlighted in gray. (DOCX 15 kb) [file 40359_2018_272_MOESM5_ESM.docx]

**S5 Table. Results for caregiver groups for the questionnaire for purpose and meaning in life (LEBE).**

|  | Specialized units  N=42 | | | At home care  N=30 | | |
| --- | --- | --- | --- | --- | --- | --- |
|  |  | *M* | *SD* |  | *M* | *SD* |
| Main scales |  |  |  |  |  |  |
| Meaning in life |  | 52.36 | 9.66 |  | 51.93 | 12.35 |
| Crisis of meaning |  | 59.95 | 10.19 |  | 56.83 | 8.33 |
| Primary scales |  |  |  |  |  |  |
| Social engagement |  | 51.98 | 10.96 |  | 52.93 | 11.72 |
| Explicit religiosity |  | 51.45 | 7.94 |  | 50.77 | 10.03 |
| Closness to nature |  | 51.64 | 12.31 |  | 48.63 | 11.85 |
| Self-knowledge |  | 53.45 | 10.23 |  | 49.73 | 11.33 |
| Health |  | 49.79 | 9.00 |  | 49.80 | 8.85 |
| Generativity |  | 52.19 | 8.83 |  | 47.80 | 12.20 |
| Spirituality |  | 51.74 | 10.58 |  | 51.70 | 10.82 |
| Challenge |  | 49.74 | 11.23 |  | 47.73 | 11.60 |
| Individualism |  | 49.38 | 10.88 |  | 43.10 | 13.99 |
| Power |  | 49.69 | 11.77 |  | 48.53 | 13.53 |
| Development |  | 49.69 | 11.94 |  | 48.27 | 12.91 |
| Performance |  | 52.00 | 10.42 |  | 47.30 | 11.84 |
| Freedom |  | 53.52 | 10.49 |  | 48.27 | 11.22 |
| Knowledge |  | 52.60 | 11.89 |  | 52.77 | 10.19 |
| Creativity |  | 52.50 | 10.58 |  | 49.07 | 11.68 |
| Tradition |  | 52.64 | 9.46 |  | 52.00 | 8.41 |
| Down-to-earth attitude |  | 52.90 | 12.51 |  | 52.33 | 9.98 |
| Moral |  | 51.71 | 10.55 |  | 51.53 | 10.59 |
| Rationality |  | 53.21 | 10.61 |  | 52.83 | 10.51 |
| Community |  | 51.29 | 10.20 |  | 50.57 | 11.42 |
| Fun |  | 50.43 | 11.42 |  | 49.40 | 15.36 |
| Love |  | 48.52 | 12.21 |  | 48.23 | 14.33 |
| Wellness |  | 45.24 | 10.35 |  | 44.93 | 13.59 |
| Wellfare |  | 52.95 | 10.31 |  | 50.41 | 11.62 |
| Conscious experience |  | 52.62 | 12.66 |  | 47.30 | 15.23 |
| Harmony |  | 44.45 | 11.81 |  | 43.50 | 11.14 |

Note: Scores between < 45 and > 55 are considered as noteworthy. Displayed are mean (M) and standard deviation (SD). Deviations from the norm are highlighted in gray.
